# Prokaryotic Community Succession in Bulk and Rhizosphere Soils Along a High-Elevation Glacier Retreat Chronosequence on the Tibetan Plateau

**DOI:** 10.3389/fmicb.2021.736407

**Published:** 2021-10-08

**Authors:** Jinbo Liu, Weidong Kong, Pinhua Xia, Chunmao Zhu, Xiangzhen Li

**Affiliations:** ^1^Department of Hepatobiliary Surgery, The Affiliated Hospital of Southwest Medical University, Luzhou, China; ^2^Academician (Expert) Workstation of Sichuan Province, The Affiliated Hospital of Southwest Medical University, Luzhou, China; ^3^Key Laboratory of Alpine Ecology, Institute of Tibetan Plateau Research, Chinese Academy of Sciences, Beijing, China; ^4^Guizhou Key Laboratory for Mountainous Environmental Information and Ecological Protection, Guizhou Normal University, Guiyang, China; ^5^Research Institute for Global Change, Japan Agency for Marine–Earth Science and Technology (JAMSTEC), Yokohama, Japan; ^6^Key Laboratory of Environmental and Applied Microbiology, Chengdu Institute of Biology, Chinese Academy of Sciences, Chengdu, China

**Keywords:** prokaryote, deglaciated soil, alpine ecology, microbial community composition, Tibetan Plateau

## Abstract

Early colonization and succession of soil microbial communities are essential for soil development and nutrient accumulation. Herein we focused on the changes in pioneer prokaryotic communities in rhizosphere and bulk soils along the high-elevation glacier retreat chronosequence, the northern Himalayas, Tibetan Plateau. Rhizosphere soils showed substantially higher levels of total organic carbon, total nitrogen, ammonium, and nitrate than bulk soils. The dominant prokaryotes were Proteobacteria, Actinobacteria, Acidobacteria, Chloroflexi, Crenarchaeota, Bacteroidetes, and Planctomycetes, which totally accounted for more than 75% in relative abundance. The dominant genus *Candidatus Nitrososphaera* occurred at each stage of the microbial succession. The richness and evenness of soil prokaryotes displayed mild succession along chronosequene. Linear discriminant analysis effect size (LEfSe) analysis demonstrated that Proteobacteria (especially Alphaproteobacteria) and Actinobacteria were significantly enriched in rhizosphere soils compared with bulk soils. Actinobacteria, SHA_109, and Thermoleophilia; Betaproteobacteria and OP1.MSBL6; and Planctomycetia and Verrucomicrobia were separately enriched at each of the three sample sites. The compositions of prokaryotic communities were substantially changed with bulk and rhizosphere soils and sampling sites, indicating that the communities were dominantly driven by plants and habitat-specific effects in the deglaciated soils. Additionally, the distance to the glacier terminus also played a significant role in driving the change of prokaryotic communities in both bulk and rhizosphere soils. Soil C/N ratio exhibited a greater effect on prokaryotic communities in bulk soils than rhizosphere soils. These results indicate that plants, habitat, and glacier retreat chronosequence collectively control prokaryotic community composition and succession.

## Introduction

Glaciers cover ∼10% of the land surface of the Earth and are rapidly shrinking in most parts of the world, leading to significant impacts on terrestrial ecosystems (Jain, 2014; Milner et al., 2017). The biological, physical, and chemical characteristics of the deglaciated soil are closely linked to the deglaciation chronosequence (Bernasconi et al., 2011). These newly exposed substrates represent natural laboratories to study primary succession of the microbial community and the concomitant development of new soil (Schuette et al., 2010). Deglaciated soil typically has low nutrient status and an absence of organic carbon (C) (Strauss et al., 2009). Microbial succession is highly correlated with soil C and nitrogen (N) contents along the deglaciation chronosequence (Zumsteg et al., 2012), and the microbial communities are the main drivers that build the soil organic matter pool, expediting pedogenesis for ecosystem succession (Sun et al., 2016). Early soil formation processes should be related to the composition of the microbial communities, the primary substrate structure, and available water (Górniak et al., 2017). The establishment of pioneering microbial communities is the key determinant of deglaciated soil development and its ecosystem function and stability (Schmidt et al., 2008; Kabala and Zapart, 2012) and facilitates the colonization of pioneering plants (Bradley et al., 2014). In a high Arctic glacier forefield, genomic data analysis showed that bacteria derived from the glacial environment was the dominant initial microbial community; a mixed community of autotrophic and heterotrophic bacteria was hosted in older soils (Bradley et al., 2016). In a high-Andean chronosequence, photosynthetic bacteria, and N-fixing bacteria, such as cyanobacteria, play important roles in the acquisition of nutrients and ecological succession in recently deglaciated soils (Schmidt et al., 2008). Cyanobacterial diversity and evenness increase in young deglaciated soils (<6 years old) before becoming stable along a chronosequence of the Tibetan Plateau (Liu et al., 2016).

Plants, during primary succession, and microbial activity in rhizosphere soil can be limited by N (Castle et al., 2017); in the case of the latter, it was found this limitation could last for 123 years in the Hailuogou Glacier forefield (Li et al., 2020). However, the initial microbial community of newly exposed soils, including those of the High Arctic, can change rapidly, suggesting that some key soil processes, such as C cycling, can also shift within a relatively short period after rapid glacial retreat (Yoshitake et al., 2018). Specific rhizobacterial communities can be selected by pioneer plants of different species in high mountain ecosystems during early primary succession (Ciccazzo et al., 2014). The stage of soil development modulates rhizosphere effect along a high Arctic desert chronosequence (Mapelli et al., 2018). Additionally, it has been found that plant–microbe interactions are important as a driver of community assembly and ecosystem succession (Bueno de Mesquita et al., 2017; Knelman et al., 2018).

The climate-induced glacier melting revealed a primary ecological succession (Cazzolla Gatti et al., 2018). Studying the succession of deglaciated soil microbial communities is important to fully understand the impact of climate change on soil system stability in alpine areas. The community structures of glacier foreland soils are strongly correlated with climatic, vegetation, and soil properties and thus closely mirror the complexity and small-scale heterogeneity of alpine soils (Donhauser and Frey, 2018). The Tibetan Plateau, with an average elevation of over 4,000 m above sea level (a.s.l.) and an area of 2.5 × 10^6^ km^2^, is the highest and most extensive highland in the world and has been named “the Third Pole” (Kang et al., 2010). Tibetan Plateau glaciers have exhibited rapid retreat due to climate warming after the little ice age (extended from AD 1400 to AD 1700) (Mann et al., 2009) and particularly since the 1980s (Yao et al., 2012). For the last years, several studies have been focused on the melt glacier foreland bulk soil community, such as an autotrophic community in Zhadang Glacier (30°28.540′N, 90°38.362′E, 5,200 m at glacier termini) (Liu et al., 2016), a bacterial community in the foreland of Baishui Glacier No. 1 (27°06′16″ N, 100°11′44″E, 4,395 m at glacier termini) (Sajjad et al., 2021), a bacterial and fungal community in Hailuogou Glacier (29°34′N, 102°00′E, 2,951 m at glacier termini) (Bai et al., 2019, 2020; Jiang et al., 2019; Li et al., 2020), and a bacterial community in Muztag Ata Glacier (38°16′N, 75°0′E, 4,350 m at glacier termini) (Khan et al., 2020). These studies were not enough as yet to understand prokaryotic community succession and the effect of plants on soil microorganism in this region. These studies were mainly focused on the bacterial succession in bulk soils from glacier forelands, few studies took into account the role of plants. These glaciers were in different altitudes and spread out in different areas of the Tibetan Plateau with different retreat times. Altitude is a sensitive environment selector of plant growth (Ma et al., 2010), not to mention the extreme oligotrophic environment of the new terrestrial habitats. Research on the high-elevation glacier retreat area is rare, and the plant effect is not clear. We hypothesized that (1) the high-elevation glacier foreland may have a special prokaryotic community structure, and (2) the pioneer plants appearing in a high-elevation glacier retreat area may be favorable for special microbes and have an effect on the microbial community structure and soil nutrient accumulation along the chronosequence.

Qiangyong Glacier is a high-elevation glacier (the terminus is at an altitude of 5,000–5,100 m) located on the northern Himalayas, Tibetan Plateau. New terrestrial habitats have emerged, and a primary succession has developed in the retreat area after the glacier retreated. Herbs and shrubs appeared along the successional chronosequence. Recently, two reports showed the bacterial community succession in Qiangyong Glacier terminus to the downstream water (Kong et al., 2019; Gu et al., 2021); the prokaryotic community in this glacier foreland and the plant effects were still unknown. To test the hypotheses addressed above, both bulk and rhizosphere soils during different succession stages were collected in Qiangyong Glacier. Primer sets specifically designed to target part of the 16S rRNA gene were used to explore the structure, diversity, and succession of prokaryotic communities in deglaciated soils along a ∼259-year chronosequence. Soil physicochemical properties (C, N, and water content) were measured, and attempts were made to identify which factor may be the key one in community succession.

## Materials and Methods

### Study Site and Soil Sampling

**FIGURE 1:**
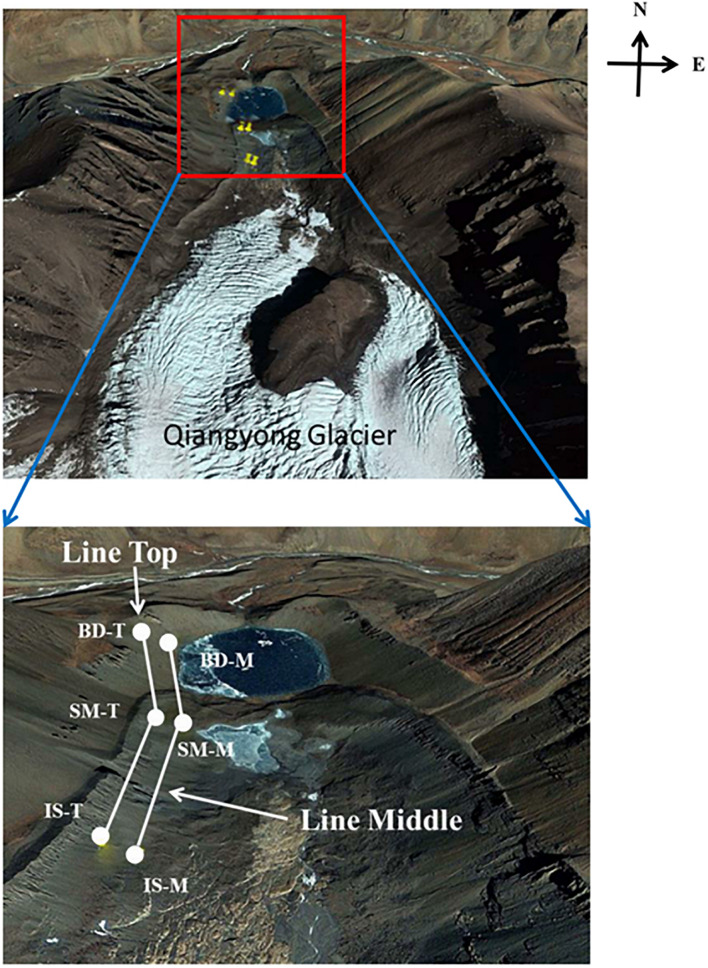
Map of sampling locations along the Qiangyong Glacier retreat chronosequence. Sites: IS, middle of glacier terminal and small Qiangyong lake; SM, midway along the edge of the smaller lake; BD, approximately one-third of the way along the edge of the larger lake; M and T, the sampling lines, Middle and Top, respectively.

Qiangyong Glacier is located on the north side of the Himalayas, with a length of 4.6 km, a maximum width of 2.8 km, and an area of 7.7 km^2^ (Luo et al., 2003). It is a continental glacier; the snow line is at an altitude of 5,600 m, and the terminus is at an altitude of 5,000–5,100 m (Li et al., 2012). From 1976 to 2006, the glacier has retreated at an average rate of 4 m year^–1^ (Yao et al., 2012). In July 2013, soil samples were collected from three sample sites (IS, SM, and BD) along two transects [line middle (LM) and line Top (LT)], on the east-facing side of the glacier movement residual moraine ridges (Figure 1). The three sample sites were as follows: the middle of the glacier termini and small Qiangyong lake upstream edge (IS), midway along the edge of the smaller lake (SM), and approximately two-thirds of the way along the edge of the larger lake (BD). At the time of sampling, sites IS_M(96a), SM_M(160a), and BD_M(259a) corresponded to a time of exposure after ice melting of 96, 160, and 259 years, respectively; sites IS_T(92a), SM_T(159a), and BD_T(258a) corresponded to a time of exposure after ice melting of 92, 159, and 258 years, respectively. The main plant located at IS and SM was *Kobresia homilies*, which then transitioned *Potentilla fruticose* at the BD sites. Bulk soil not in contact with the root system and located at 50–100 cm from each sampled abundant plant was collected from 3 to 5 cm after removing the top 1–2 cm of large sand grains. Soil samples near the plant root (less than 1 cm) were collected as rhizosphere soil from a similar depth as the bulk soils. Soil samples were stored in sterile sampling bags (Labplas, Canada) and transferred to the laboratory on ice. One part of each sample was stored at −80°C until DNA extraction was performed; the other part was used for physicochemical property analyses.

### Soil Physicochemical Property Determination

Soil factors were measured using the methods described in Guo et al. (2015) and will only be summarized here. Soil total C and total organic C (TOC) were measured in the solid state using a TOC analyzer (TOC-L, Shimadzu, Japan); soil total nitrogen (TN) was determined by elemental analyzer (vario MAX, Elementar, Germany). Soil nitrate (NO_3_^–^-N) and ammonium (NH_4_^+^-N) were extracted with 1 M KCL and determined using an Automated Discrete Analyzer (AQ2, SEAL Analytical Inc., England). Soil water content (WC) was gravimetrically determined after drying at 105°C for 12 h.

### Sample DNA Extractions, PCR Amplification, and High-Throughput Sequencing

Soil genomic DNA was extracted from 0.5 g of frozen soil using the FastDNA^®^ spin kit for soil (MP Biomedicals, Solon, OH, United States) following the manufacturer’s protocol. The quantity of the DNA was determined using a NanoDrop ND-1000 spectrophotometer (NanoDrop Technologies Inc., Wilmington, DE, United States). The primers 515F (5′-GTG YCA GCM GCC GCG GTA-3′) and 806R (5′-GGA CTA CHV GGG TWT CTA AT-3′) were used for Illumina MiSeq sequencing at the Chengdu Institute of Biology, Chinese Academy of Sciences. PCR amplification and high-throughput sequencing method was according to Li et al. (2015).

### Sequence Analysis

The raw sequences were sorted based on the unique sample barcodes, and then quality control was performed for the sequences using QIIME pipeline (Caporaso et al., 2010). All sequence reads were trimmed and assigned to each sample based on their barcodes. The sequences with high quality (length > 150 bp, without ambiguous base “N,” and average base quality score > 30) were used for downstream analysis. Chimeric sequences were identified and removed using the Uchime algorithm (Edgar et al., 2011). After filtering and chimera removal, *de novo* operational taxonomic unit (OTU) picking was performed using uclust at 97% sequence identity, and subsequently, taxonomy was assigned to OTU based on the Greengenes database at a confidence level of 80% (version 13.8). All low-abundance Archaeal and Bacterial sequences that were only detected in one of the samples were culled. To avoid the influence of sequencing depth, rarefaction was performed with 9,000 sequences per sample for the diversity of microorganisms.

### Statistical Analyses

Community α-diversity was estimated using Shannon, Evenness, and Richness diversity indices. The β-diversity was calculated based on Bray–Curtis distances, and the differences in community structure between samples were visualized using principal coordinates analysis (PCoA) plot. Paired-samples *t*-tests were used for comparing data of bulk and rhizosphere soil with the SPSS 18.0 statistical software package. Adonis analysis was used to compare the difference between groups, and redundancy analysis (RDA) based on Bray–Curtis distances was used to determine the most significant environmental variables that might influence the prokaryotic community structure using the vegan and picante packages of the statistical platform R. Linear discriminant analysis (LDA) effect size (LEfSe) analyses between different sample groups utilized an online platform (Segata et al., 2011) and was applied to the OTU table to identify discriminant prokaryotic taxa.

### Data Submission

The raw sequence reads generated in the present study are available in the National Center for Biotechnology Information (NCBI) Sequence Read Archive under the project ID PRJNA595717.

## Results

### Alpine Plants Altered the Accumulation of Soil Carbon and Nitrogen Content Along the Chronosequence

In total, the contents of TOC, TN, NH_4_^+^-N, and NO_3_^–^-N in the rhizosphere were all significantly higher than in bulk soil (*n* = 18, *p* < 0.01). The TOC and TN contents showed an increasing trend along the glacier chronosequence, while NH_4_^+^-N and NO_3_^–^-N contents showed a decreasing trend, at line middle and line top, respectively (Supplementary Figure 1).

### Alpine Plant Effects on Prokaryotic Community Composition and Succession

**FIGURE 2:**
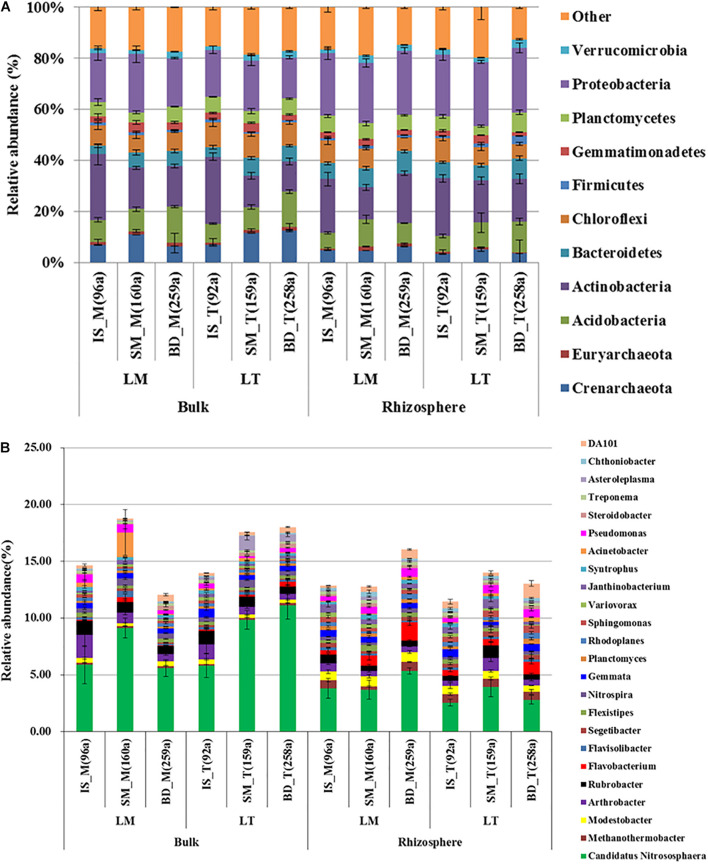
The relative abundance of prokaryotic phyla **(A)** and genera (**B**). Sites: IS, middle of glacier terminal and small Qiangyong lake; SM, midway along the edge of the smaller lake; BD, approximately one-third of the way along the edge of the larger lake; M and T, the sampling lines, Middle and Top, respectively; S and R, bulk and rhizosphere soils, respectively. The relative abundances are given as a percentage of each phylum or genus against the total 16S rRNA gene sequences. Only phyla with average relative abundance > 1% are shown. Only genera with average relative abundance > 0.18% are shown. Data are shown as average ± SE (*n* = 3).

In the phyla and genus level community composition, there were some clades detected in different abundance and succession trends between bulk and rhizosphere soil. In the phylum level, the dominant phyla (relative abundance > 5%) across all samples were Proteobacteria (21.95%), Actinobacteria (18.11), Acidobacteria (9.50%), Chloroflexi (7.9%), Crenarchaeota (6.81%), Bacteroidetes (6.27%), and Planctomycetes (5.69%), accounting for more than 75% of the total prokaryotic sequences. The relative abundance of the phyla Gemmatimonadetes, Verrucomicrobia, Euryarchaeota, and Firmicutes were > 1% (Figure 2A). The relative abundances of Proteobacteria and Bacteroidetes in rhizosphere soil was significantly higher than that in bulk soil; Crenarchaeota and Gemmatimonadetes were on the opposite (*n* = 36, *p* < 0.01). In bulk soil, the relative abundance of Acidobacteria showed an increasing trend along the chronosequence, while Actinobacteria displayed the opposite trend. A similar trend was also found in rhizosphere soil of the top line samples.

The dominant genera (average relative abundance > 0.5%) across all samples were *Candidatus Nitrososphaera* (5.7%), *Arthrobacter* (0.83%), *Rubrobacter* (0.78%), *Modestobacter* (0.55%), *Flavobacterium* (0.53%), *Pseudomonas* (0.53%), and *Gemmata* (0.52%). Seventeen other genera were found in the majority of samples with an average relative abundance of more than 0.18% (Figure 2B). Among them, the relative abundance of the predominant genus *Candidatus Nitrososphaera* in rhizosphere soil (3.69%) was significantly lower than that in bulk soil (7.89%) (*n* = 36, *p* < 0.001). The relative abundance of *Modestobacter* and *Flavobacterium* in rhizosphere soil was significantly higher than in bulk soil (*n* = 36, *p* < 0.001, *p* < 0.05). The relative abundances of *Rubrobacter* was on the opposite (*n* = 36, *p* < 0.05). Flavobacterium has an increased trend along the chronosequence. Modestobacter was decreased from site IS to SM and then increased from SM to BD in bulk soil, while in the rhizosphere they were on the opposite trend. *Rubrobacter* was relatively stable from site IS to SM, and then decreased from site SM to BD in bulk soil but increased in rhizosphere soil.

### Alpine Plants Altered Prokaryotic Community α-Diversity Succession Along the Chronosequence

**FIGURE 3:**
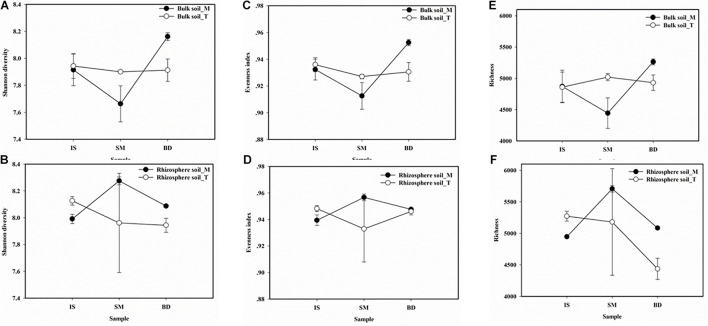
Prokaryotic community α-diversity succession along the chronosequence. **(A)** and **(B)** were Shannon diversity in bulk and rhizoshpher soils, respectively. **(C)** and **(D)** were the Evenness index in bulk and rhizoshpher soils, respectively. **(E)** and **(F)** were the Richness in bulk and rhizoshpher soils, respectively. Data were shown as average ± se (*n* = 3). Sites: IS, middle of glacier terminal and small Qiangyong lake; SM, midway along the edge of the smaller lake; BD approximately one-third of the way along the edge of the larger lake. M and T indicate the sampling lines, Middle and Top, respectively.

Prokaryotic community α-diversity indices showed mild succession in the glacier retreat chronosequence (Figure 3). In total, the averages of the three indices in the rhizosphere soil were slightly higher than those in bulk soils. In bulk soils, the indices were decreased from site IS to SM and then increased from SM to BD; the final value in BD was higher than that in IS in the line Top sample (Figures 3A,C,E). The opposite trend of succession was found in the rhizosphere soil in the line Top sample (Figures 3B,D,F). In line middle, the richness in bulk soil was relatively stable in bulk soil, but that has a drop in the rhizosphere soil, especially at site BD (Figures 3E,F).

### Alpine Plant Effects on Prokaryotic Community β-Diversity

**FIGURE 4:**
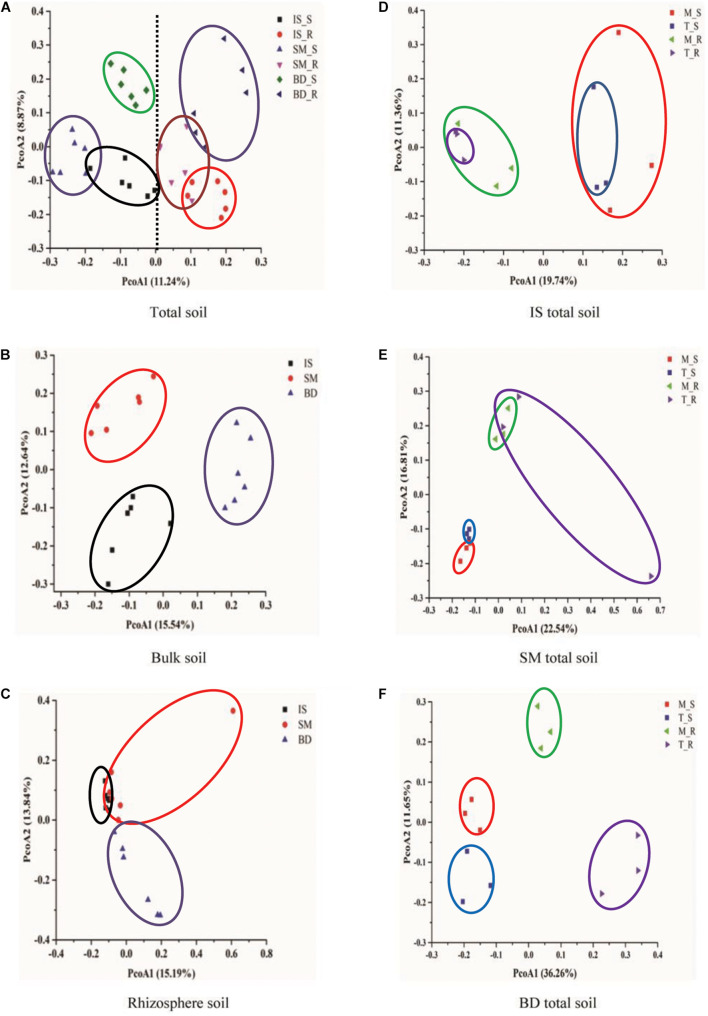
PCoA plot of soil bacterial community composition. **(A)** For total soil, **(B)** for bulk soil, **(C)** for rhizosphere soil, **(D)** for IS total soil, **(E)** for SM total soil, and **(F)** for BD total soil. Sites: IS, middle of glacier terminal and small Qiangyong lake; SM, midway along the edge of the smaller lake; BD approximately one-third of the way along the edge of the larger lake. M and T indicate the sampling lines, Middle and Top, respectively; S and R represent bulk and rhizosphere soils, respectively.

Alpine plants affect the prokaryotic community β-diversity (Figure 4). Bulk and rhizosphere soils separated well from each other along the first axis (Figure 4A). This was in agreement with the Adonis analysis, which showed that the community composition of bulk and rhizosphere soils were significantly different (*n* = 18, *p* < 0.001). In bulk soil, each of the sampling sites (IS, SM, and BD) grouped separately (Figure 4B). In rhizosphere soil, the IS samples were close to SM along the positive direction of the second PCoA axis, but two subgroups were found in the BD samples, one of which (three samples all from the middle line) was much closer to the other two sampling sites (Figures 4C,E,F). According to Adonis analysis, the community compositions of IS, SM, and BD were significantly different in total, bulk, and rhizospheres soils, respectively (*n* = 12, 6, 6, *p* < 0.001). For each of the three sample locations (IS, SM, and BD), Adonis analysis showed that the bulk and rhizosphere soils within the same site were significantly different from each other (*n* = 6, *p* < 0.01) (Figures 4D–F). From IS to BD, the distance between the group centroids of the middle and top lines showed an increasing trend and no significant difference (*n* = 6, *p* = 0.576, 0.314, and 0.053, respectively) (Figures 4D–F). Together, these results suggested that the prokaryotic community composition was different at all three locations.

### Special Selection of Microorganisms by Alpine Plants Along the Chronosequence

**FIGURE 5:**
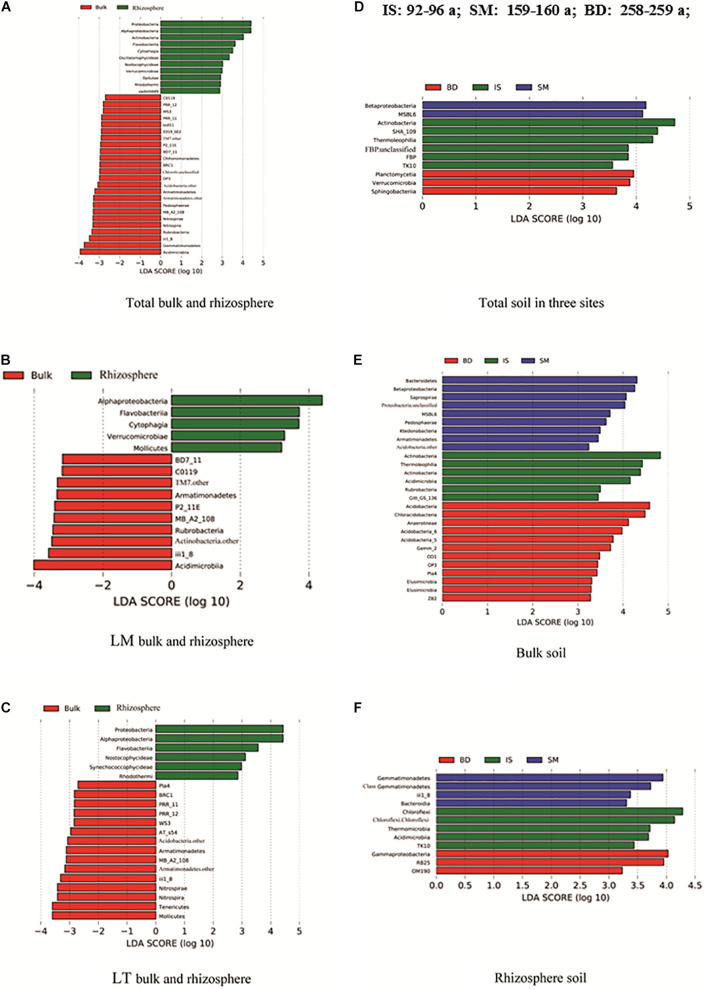
LEfSe analyses of different sample groups. **(A)** For tobal bulk and rhizosphere soil, **(B)** for LM bulk and rhizosphere soil, **(C)** for LT bulk and rhizosphere soil, **(D)** for total soil in three sites, **(E)** for bulk soil, and **(F)** for rhizosphere soil. Sites: IS, middle of glacier terminal and small Qiangyong lake; SM, midway along the edge of the smaller lake; BD approximately one-third of the way along the edge of the larger lake. LM and LT indicate the sampling lines, Middle and Top, respectively; the different colors in the LE analysis represent different groups. The closer the sample distance is, the more similar the microbial compositions of the samples are, and the smaller the difference is (*P* ≤ 0.01).

Across the glacier successional stages, different clades were detected in the bulk and rhizosphere soil fractions, which explained the statistically significant differences between their respective microbial communities (Figure 5). The numbers of discriminant clades were 24 and 11 from bulk and rhizosphere soils, respectively (Figure 5A). The number of discriminant clades increased in bulk soils from site IS to BD (6, 9, and 12), while the opposite trend was observed in rhizosphere soils (5, 4, and 3) (Figures 5E,F). In general, the phyla Proteobacteria, especially class Alphaproteobacteria, and Actinobacteria were significantly enriched in rhizosphere soil; meanwhile, Acidimicrobiia and Gemmatimonadetes were significantly enriched in bulk soil (Figure 5A). The number of discriminant clades in line top bulk and rhizosphere soils were both higher than in the respective line middle soils. In line middle, Alphaproteobacteria were significantly enriched in the rhizosphere soil, while Acidimicrobiia were enriched in bulk soil (Figure 5B). In line top, the phylum Proteobacteria, and specifically the class Alphaproteobacteria, was significantly enriched in rhizosphere soil. Meanwhile, Tenericute and Mollicutes were more enriched in bulk soil (Figure 5C). Among three sample sites in the total sample, site IS was enriched with Actinobacteria, SHA_109, and Thermoleophilia. Site SM was enriched with Betaproteobacteria and OP1.MSBL6. Site BD was enriched with Planctomycetia and Verrucomicrobia (Figure 5D).

### Effect of Environmental Factors of Microbial Community Composition

**FIGURE 6:**
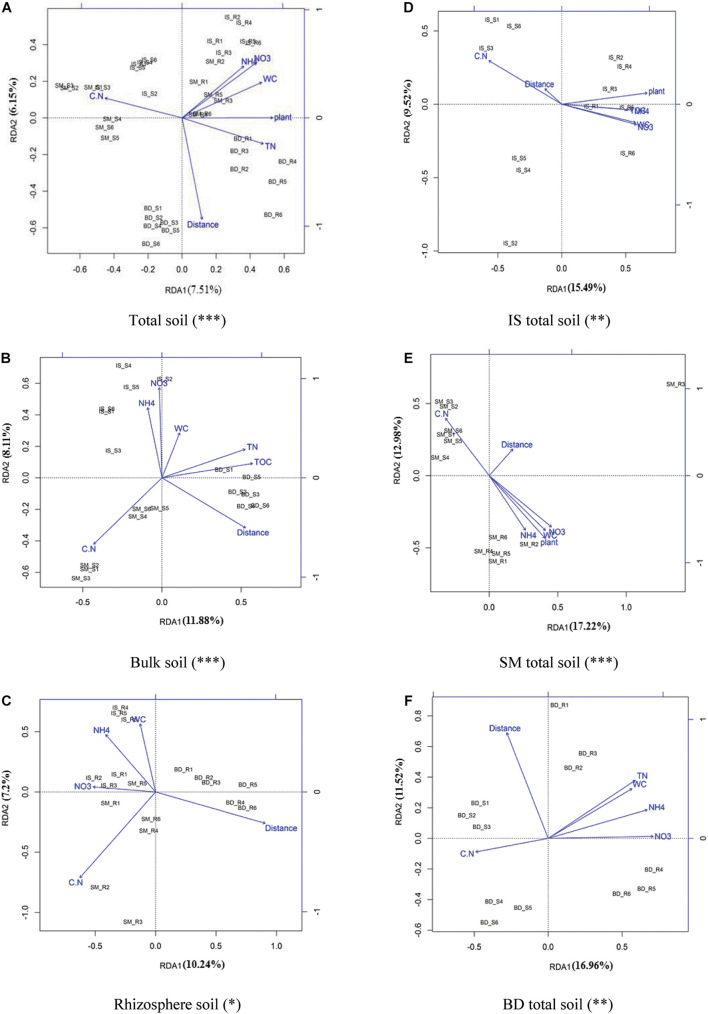
Bacterial community and environment factor RDA analysis. **(A)** For total soil, **(B)** for bulk soil, **(C)** for rhizosphere soil, **(D)** for IS total soil, **(E)** for SM total soil, and **(F)** for BD total soil. Sites: IS, middle of glacier terminal and small Qiangyong lake; SM, midway along the edge of the smaller lake; BD approximately one-third of the way along the edge of the larger lake. Significance: ***0.001; **0.01; and *0.05. C:N, total Carbon to total Nitrogen ratio; WC, water content.

RDA analysis was used to assess which factors affected the microbial community structure of the samples (Figure 6). In total, eight factors were detected: TOC, TN, NH_4_^+^-N, NO_3_^–^-N, C/N ratio, WC, plants, and distance. For total soil samples, all factors except TOC had significant impact on the prokaryotic community structure (*n* = 36, *p* < 0.001) (Figure 6A). Among them, all the rhizosphere soils were significantly affected by plants. Distance affects both bulk and rhizosphere soil prokaryotic community structure; its effect on different samples sites was in the order of BD > SM > IS. The C/N affected the bulk soil microbial community structure more than that of the rhizosphere soil, while NH_4_^+^-N, NO_3_^–^-N, and WC had the opposite trend (Figures 6A,D–F). In bulk soil, all factors except plants had significant effect on the prokaryotic community structure (*n* = 18, *p* < 0.001); the effect of C/N from different sample sites was in the order of SM > IS > BD (Figure 6B). In rhizosphere samples, NH_4_^+^-N, NO_3_^–^-N, C/N ratio, WC, and distance were the main factors affecting the prokaryotic community structure (*n* = 18, *p* < 0.05); the effect of NH_4_^+^-N, NO_3_^–^-N, and WC from different sample sites was in the similar order of IS > SM > BD (Figure 6C).

## Discussion

### A Special Prokaryote Community Structure Has Been Found in the High-Elevation Glacier Chronosequences

As expected, the special prokaryote community structures in the high-elevation Qiangyong Glacier foreland soil were revealed in this study. The relative abundance of seven phyla was above 5%; in a total of 11 phyla the relative abundance was above 1% (Figure 2A). The number of phyla was higher than that found in the frozen soil [five most abundant phyla (>2%)] from the glacier of the northwestern Himalayas of Jammu and Kashmir, India (Gupta et al., 2020); the two studies both were conducted in Himalayas. Furthermore, the three top abundant phyla detected in this research were also found in other glacier forelands of the Tibetan Plateau but with different order and abundance. For example, Proteobacteria (43%) and Actinobacteria (16%) were the predominant bacteria phyla in soils from Hailuogou Glacier retreat region. Similarly, they were also reported on the Muztag Ata Glacier chronosequence (Khan et al., 2020) and the frontier of Baishui Glacier No.1 (Sajjad et al., 2021). In this research, the Proteobacteria average relative abundance was 21.95% in total and comparable to the data from Gupta’s report (Gupta et al., 2020). The reason may be the effect of elevation, as in this research the glacier termini were at the elevation of 5,000–5,100 m; the height of the glacier is 4,700 m a.s.l. in Gupta’s report. The glaciers in other reports cited above were all below 4,400 m (Sun et al., 2016; Khan et al., 2020; Sajjad et al., 2021). These results suggested that as the elevation increased, the abundance of Proteobacteria decreased; at the same time, other phyla were enriched. As the microbial community reports on high-elevation glacier foreland were few, further research in this area is being looked forward to.

### Plant Rhizosphere Selected Specific Prokaryotic Communities and Shifted Nutrient Accumulation Along the Qiangyong Chronosequence

The relative abundance of Proteobacteria, Bacteroidetes, *Modestobacter*, and *Flavobacterium* in rhizosphere soil was significantly higher than that in bulk soil; some genera were in the opposite succession trend compared with bulk and rhizosphere soil (Figure 2). The LEfse detected differential clades infractions, which consistently explained the statistically significant difference between the bulk soil and rhizosphere soil prokaryotic communities (Figure 5). In general, the phyla Proteobacteria, especially class Alphaproteobacteria, and Actinobacteria were significantly enriched in rhizosphere soil; meanwhile, Acidimicrobiia, and Gemmatimonadetes were significantly enriched in bulk soil. At three sites, bulk and rhizosphere soil also had enriched clades. This result was different from reports found for low elevation, such as the finding that rhizobacterial communities were mainly composed of Acidobacteria and Proteobacteria, whereas bare soil was colonized by Acidobacteria and Clostridia in the foreland of Weisskugel Glacier (2,400 m a.s.l.) (Ciccazzo et al., 2014).

Meanwhile, in this research, the detected nutrient contents in rhizosphere soil were significantly higher than in bulk soil (Supplementary Figure 1). The α-diversity in rhizosphere soil was higher than in bulk soil (Figure 3); PCoA and Adonis analysis showed the bulk and rhizosphere soil microbial community was significantly different along the chronosequence (Figure 4). Based on the RDA analysis, plants also showed a significant effect on microbial community structures in Qiangyong Glacier foreland (Figure 6). These results suggest that the plants altered the soil C and N accumulation by shifting the soil prokaryotic community structure. This consisted of the most enriched phyla in rhizosphere soil (phyla Proteobacteria, especially class Alphaproteobacteria, and Actinobacteria) (Figure 5). Proteobacteria were detected as autotrophic microbial communities in Qiangyong Glacier-originated water (Kong et al., 2019). They were also supposed to have the ability for N fixation at a glacial foreland on Anvers Island (Strauss et al., 2012). Actinobacteria and Betaproteobacteria have the potential to photosynthesize; Betaproteobacteria and Alphaproteobacteria have the potential to perform nitrification and denitrification of microbes from supraglacial cryoconite of polar regions (Cameron et al., 2012). Furthermore, the dominance of Actinobacteria in Antarctic soils has been linked to specific trace elements (magnesium, calcium, and potassium) and salts in the associated glacier forelands (Bajerski and Wagner, 2013). Actinobacteria are adapted to oligotrophic environments where their hyphae allow them to restore nutrients and moisture through pores in the soil (Arocha-Garza et al., 2017; Zhang et al., 2019). The rhizosphere soil microbial development significantly affected soil organic C and total N accumulation in the Hailuogou Glacier forefield (Li et al., 2020). The results above suggest that the emergence of plants had a selective effect on microorganisms and promoted the accumulation of C and N in this high-elevation glacier retreat in rhizosphere soils.

### Environmental Parameters Related to Pedogenesis Shape the Prokaryotic Communities in Bulk and Rhizosphere Soils

The concentration of key nutrients related to soil fertility changed from site IS to BD along the Qiangyong chronosequence (Supplementary Figure 1). All detected environment parameters had effects on the high-elevation prokaryotic community composition succession (Figure 6). This result means that the distance, plant, and soil physicochemical properties together determine microbial community structures in the Qiangyong Glacier foreland. This result was partly similar to the reports of others. Microbial community structure is strongly conditioned by the successional stage, deglaciation time, water content, plants, spruce leachate, and the C and N contents of the foreland soil (Tscherko et al., 2004; Noll and Wellinger, 2008; Göransson et al., 2011; Knelman et al., 2012, 2018; Górniak et al., 2017; Kim et al., 2017; Bai et al., 2020). Most of the above reports did not include rhizosphere soil, and these studies were on elevations of < 1,000 m (Knelman et al., 2012, 2018; Kim et al., 2017), 1,780 m (Górniak et al., 2017), 1,920–2,054 m (Göransson et al., 2011), 1,950–2,050 m (Noll and Wellinger, 2008), 2,280–2,450 m (Tscherko et al., 2004), and 2,951 m at glacier termini (Bai et al., 2020). The effects of the high-elevation environmental parameters on the glacier foreland prokaryotic community were seldom found in field research. In this research, the effect of C/N on the bulk soil microbial community was greater than that in rhizosphere soil, while NH_4_^+^-N, NO_3_^–^-N, and WC had the opposite trend. The above detected different physicochemical properties in each research, and most of them were in a relatively low elevation glacier retreat area. These results suggest that the distinct habitat was not ignorable. All the above results showed that plants, habitat, and glacier retreat chronosequence collectively control prokaryotic community composition and succession.

## Conclusion

The soil TOC and TN contents showed an increasing trend along the deglaciation chronosequence, while ammonium and nitrate content showed the opposite trend. TOC, TN, ammonium, and nitrate contents in rhizosphere soil were significantly higher than in bulk soil (*p* < 0.05). In total, 11 phyla (relative abundance > 1%) and 7 dominant genera (relative abundance > 0.5%) were observed in all samples. According to LEfSe analyses, the Proteobacteria, Alphaproteobacteria, and Actinobacteria were significantly enriched in rhizosphere soil. Acidimicrobiia and Gemmatimonadetes were significantly enriched in bulk soil. No statistically significant difference was observed in the values from multiple α-diversity indices. Bacterial β-diversity showed that bulk and rhizosphere soils were obviously separate. Distance, soil C/N ratio, plants, and physicochemical properties all affected the soil bacterial community composition along the chronosequence. These results indicated that plants, habitat, and glacier retreat chronosequence collectively control prokaryotic community composition and succession.

## Data Availability Statement

The datasets presented in this study can be found in online repositories. The names of the repository/repositories and accession number(s) can be found in the article/Supplementary Material.

## Author Contributions

JL and WK contributed to conception and design of the study. PX and CZ collected the samples and determined soil physicochemical properties. JL conducted soil DNA extractions, performed the statistical analysis, and wrote the first draft of the manuscript. XL conducted the high-throughput sequencing. All authors contributed to manuscript revision and approved the submitted version.

## Conflict of Interest

The authors declare that the research was conducted in the absence of any commercial or financial relationships that could be construed as a potential conflict of interest.

## Publisher’s Note

All claims expressed in this article are solely those of the authors and do not necessarily represent those of their affiliated organizations, or those of the publisher, the editors and the reviewers. Any product that may be evaluated in this article, or claim that may be made by its manufacturer, is not guaranteed or endorsed by the publisher.
