# Supplementary material for: Prokaryotic Community Succession in Bulk and Rhizosphere Soils Along a High-Elevation Glacier Retreat Chronosequence on the Tibetan Plateau
Source: Front Microbiol. 2021 Oct 8;12:736407. doi: 10.3389/fmicb.2021.736407 (PMC8531754; doi:10.3389/fmicb.2021.736407)

Supplementary material

Fig S1 Soil physicochemical properties. (a) and (b) were Total organic in bulk and rhizoshpher soils, respectively. (c) and (d) were the total N in bulk and rhizoshpher soils, respectively. (e) and (f) were the NH_4^+-N in bulk and rhizoshpher soils, respectively. (g) and (h) were the NO_3^--N in bulk and rhizoshpher soils, respectively. IS: the middle of glacier terminal and small Qiangyong lake; SM: the middle of small lake; BD: the down one-third of big lake. M and T indicate the sampling line Middle and Top. Data was shown as average ±se (n=3). Treatment means with the same letter were not significantly different (*P*<0.05).

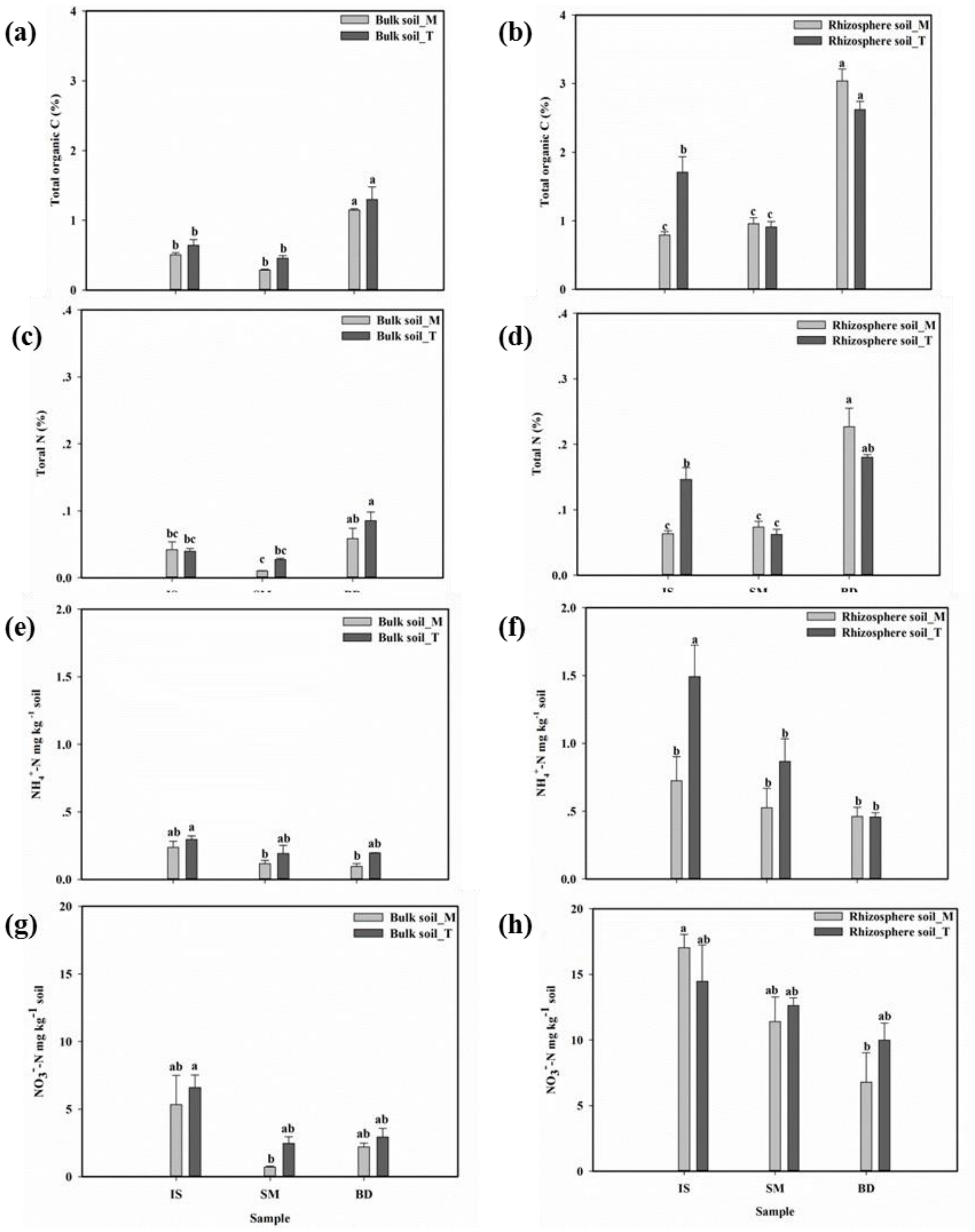

Supplement: Supplementary file 1 [file Data_Sheet_1.pdf]
